# Alterations in macrophage polarization in the craniofacial and extracranial skeleton after zoledronate application and surgical interventions – an *in vivo* experiment

**DOI:** 10.3389/fimmu.2023.1204188

**Published:** 2023-05-24

**Authors:** Ann-Kristin Struckmeier, Falk Wehrhan, Raimund Preidl, Melanie Mike, Tina Mönch, Lea Eilers, Jutta Ries, Leah Trumet, Rainer Lutz, Carol Geppert, Marco Kesting, Manuel Weber

**Affiliations:** ^1^ Department of Oral and Cranio-Maxillofacial Surgery, Friedrich-Alexander University Erlangen-Nürnberg (FAU), Erlangen, Germany; ^2^ Comprehensive Cancer Center Erlangen-European Metropolitan Area of Nürnberg (CCC ER-EMN), Erlangen, Germany; ^3^ Deutsches Zentrum Immuntherapie (DZI), Erlangen, Germany; ^4^ Department of Operative Dentistry and Periodontology, Friedrich-Alexander University Erlangen-Nürnberg (FAU), Erlangen, Germany; ^5^ Institute of Pathology, Friedrich-Alexander Universität Erlangen-Nürnberg (FAU), Erlangen, Germany

**Keywords:** osteonecrosis, bisphosphonate, macrophage, tooth extraction, MRONJ

## Abstract

**Purpose:**

Medication-related osteonecrosis occurs exclusively in the jaw bones. However, the exact pathogenesis of medication-related osteonecrosis of the jaw (MRONJ) and the unique predisposition of the jaw bones have not been elucidated, making its treatment a challenge. Recent evidence indicates that macrophages might play a pivotal role in MRONJ pathogenesis. The aim of the present study was to compare the macrophage populations between the craniofacial and extracranial skeleton and to investigate the changes induced by zoledronate (Zol) application and surgical interventions.

**Materials and methods:**

An *in vivo* experiment was performed. 120 wistar rats were randomized to 4 groups (G1, G2, G3, G4). G1 served as an untreated control group. G2 and G4 received Zol injections for 8 weeks. Afterwards, the right lower molar of the animals from G3 and G4 was extracted and the right tibia osteotomized followed by osteosynthesis. Tissue samples were taken from the extraction socket and the tibia fracture at fixed time points. Immunohistochemistry was conducted to determine the labeling indexes of CD68^+^ and CD163^+^ macrophages.

**Results:**

Comparing the mandible and the tibia, we observed a significantly higher number of macrophages and a heightened pro-inflammatory environment in the mandible compared to the tibia. Tooth extraction caused an increase of the overall number of macrophages and a shift toward a more pro-inflammatory microenvironment in the mandible. Zol application amplified this effect.

**Conclusion:**

Our results indicate fundamental immunological differences between the jaw bone and the tibia, which might be a reason for the unique predisposition for MRONJ in the jaw bones. The more pro-inflammatory environment after Zol application and tooth extraction might contribute to the pathogenesis of MRONJ. Targeting macrophages might represent an attractive strategy to prevent MRONJ and improve therapy. In addition, our results support the hypothesis of an anti-tumoral and anti-metastatic effect induced by BPs. However, further studies are needed to delineate the mechanisms and specify the contributions of the various macrophage phenotypes.

## Introduction

Medication-related osteonecrosis of the jaw (MRONJ) is a severe debilitating condition, at present mainly attributable to antiresorptive therapy (1). The prevalence ranges from 1 to 15% in cancer patients (1) and 0.02 to 0.35% in osteoporosis patients receiving antiresorptive treatment (2). The nitrogen-containing bisphosphonates (BPs) zoledronate (Zol) and alendronate are the most potent (3) and widely used antiresorptive drugs (4). BPs are the treatment of choice for osteoporosis, patients at risk of developing skeletal-related events (SRE), e.g., fractures, bone metastases or hypercalcemia in patients with advanced malignancies and multiple myeloma (5–8).

It has been proven that BPs reduce the risk of osteoporotic and metastatic fractures, bone pain, disability, and thereby improve quality of life (9, 10). Despite the great benefits of BPs and other antiresorptive medications, they have the disadvantage of potentially causing a MRONJ (11). MRONJ was first described by Marx in 2003 (12) and is defined as a nonhealing exposed bone for 8 weeks in patients with a history or ongoing use of an antiresorptive or antiangiogenic agent and no history of radiation exposure to the head and neck region (11). MRONJ is a locally destructive and devastating disease, which is associated with a significant morbidity. Due to severe complications, i.e., pathological fractures or extensive areas of infected necrotic bone, a complete resection of the necrotic bone and a microvascular reconstruction are often inevitable.

The exact pathophysiology of MRONJ is not completely understood. However, a multifactorial etiology for which many questions remain unanswered is assumed (11). The actual theory is that nitrogen-containing BPs have a high affinity to hydroxylapatite crystals in the bone and initiate apoptosis of osteoclasts by intervening in the mevalonate pathway and therefore the protein prenylation (13). Hereby, the bone density increases (14), remodeling is reduced (14) and the blood supply decreases (15).

The BP uptake in bones occurs in direct proportion to their local turnover rate. Since the jaw bones have high rates of bone remodeling, they are sites of high BP uptake and accumulation, which results in concentrations excessively inhibiting osteoclasts and thereby in decreased rates of bone remodeling (16, 17). This mechanism is enhanced in case of a local infection (18). Nevertheless, osteoclast inhibition can take place anywhere in the body and therefore the impaired osteoclastic activity cannot be the only cause of MRONJ.

In recent decades, many researchers tried to explain the pathogenesis of MRONJ: BPs were described to alter angiogenesis (19), exert infections and inflammation (20, 21), and to exert a direct toxic effect on soft tissue (22, 23). However, these theories are not entirely sufficient for the pathogenesis of MRONJ.

Increasing evidence suggests that the immunomodulatory properties of BPs contribute to the development of MRONJ as well. Hoefert et al. described a local immunosuppression and thereby a disturbance of macrophage function induced by BPs in MRONJ in contrast to other infectious jaw diseases (21).

Macrophages are a heterogeneous population of myeloid cells, which are abundant in the tissue and play crucial roles during inflammation processes (24), tissue remodeling (25), angiogenesis (26), immunoregulation (27), and tumor promotion (28, 29). They exhibit distinct functional phenotypes. Mills classified the macrophages according to their activation state into the classically-activated M1 phenotype and the alternatively-activated M2 phenotype (30). The macrophage phenotypes are characterized by different surface markers: CD68 is a pan-macrophage marker, M1 macrophages typically exhibits CD11c and the M2 phenotype highly expresses CD163, CD204, or CD206 (31–33). M1 macrophages are described to drive tissue damage through pro-inflammatory and anti-tumor activity, whereas M2 macrophages support an anti-inflammatory environment and thereby tissue regeneration (34).

In the tumor stroma, macrophages differentiate into tumor-associated macrophages (TAMs), which resemble predominantly M2 macrophages (35) and therefore, have mostly pro-tumoral effects (36). They are assumed to play an important role in tumor progression (36). Since macrophages and osteoclasts share the same lineage (37), TAMs may also be affected by BPs. E.g., it was previously described that Zol inhibits the proliferation and increases apoptosis in both osteoclasts and macrophages (38). Evidence was provided that BPs improve the survival of breast cancer patients independent of their antiresorptive effect by exerting an anti-metastatic and anti-tumoral effect (39, 40). There is evidence that TAMs might contribute to these BP-derived effects (41).

Skeletal homeostasis is dynamically influenced by the immune system. Especially, lymphocyte- and macrophage-derived cytokines were described to be some of the most potent mediators of osteoimmunoregulation (42). A well-known reason for the unique predisposition of the jaw bones for MRONJ is that the jaw bones hold teeth which are potential infection sources and are covered with only a thin mucosal membrane, which can be easily damaged and therefore can cause an infection of the underlying jaw bone (43). Moreover, Faloni et al. compared the osteoclastogenic potential of bone marrow cells between the jaw and the tibia in mice. They observed different dynamics of osteoclastogenesis comparing both types of bones and concluded that there might be functional differences between the osteoclasts, too (44). Moreover, an *in vivo* experiment showed that osteoclasts from the jaw internalize a larger amount of BPs than osteoclasts from the tibia and femur. However, the difference in BP uptake did not differentially affect osteoclastogenesis, suggesting that osteoclasts from the jaw are less sensitive to BPs after internalization (17). Differences in the composition of immune cells between the jaw, i.e., the craniofacial skeleton, and a bone belonging to the extracranial skeleton, i.e., the tibia, might be an additional reason for the unique predisposition of the jaw bones for MRONJ. However, the macrophage populations have never been compared between the craniofacial and extracranial skeleton.

The aim of this study was to investigate the macrophage polarization in the craniofacial and extracranial skeleton depending on Zol application and surgical interventions. Differences in immune cell composition may provide further insight into the pathogenesis of MRONJ and may provide an additional explanation for the unique predisposition of the jaw bones for MRONJ.

## Materials and methods

The experiment was conducted with 120 Wistar rats. It was approved by the local authorities “Regierung von Mittelfranken” No. 54-25321-3/09 and was part of the Deutsche Forschungsgesellschaft (DFG) project WE52731/1-1.

### Animals

The male, 6 months old Wistar rats were divided into 4 groups (G; G1, G2, G3, and G4).

**Figure 1:**
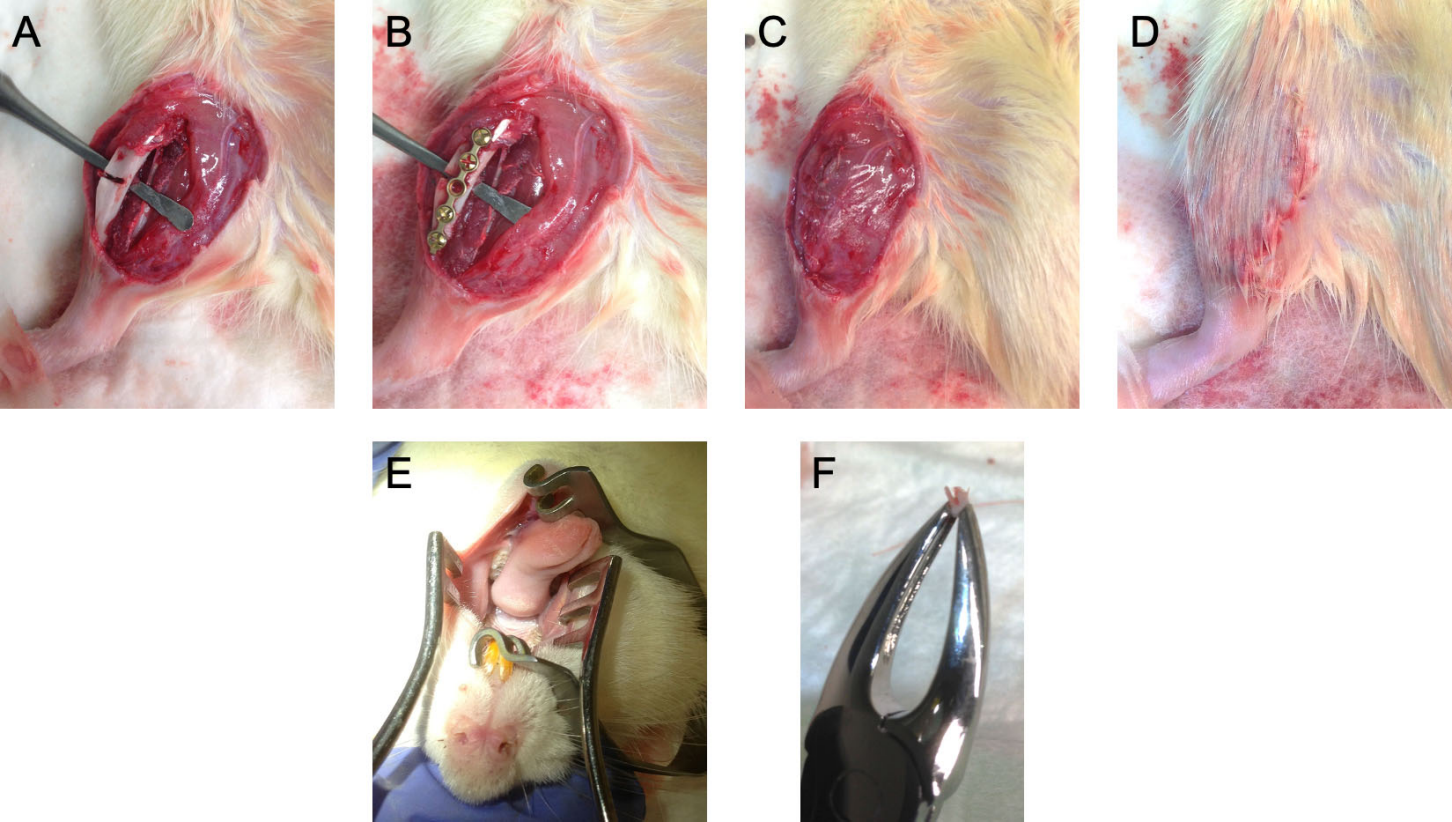
Photos of the surgical interventions. At a time point of 8 weeks, the right tibia was osteotomized followed by a microplate osteosynthesis in rats from group 3 and 4 **(A, B)**. The site of the incision was closed by multilayer suturing **(C, D)**. Additionally, the right lower molar of the animals was extracted with forceps **(E, F)**. Surgical interventions were conducted under general anesthesia using 100 mg/kg of ketamine hydrochloride (Parke-Davis, Berlin, Germany) and 2.5 mg/kg of Xylocain (Bayer, Leverkusen, Germany) *via* intraperitoneal injection.

**Figure 2:**
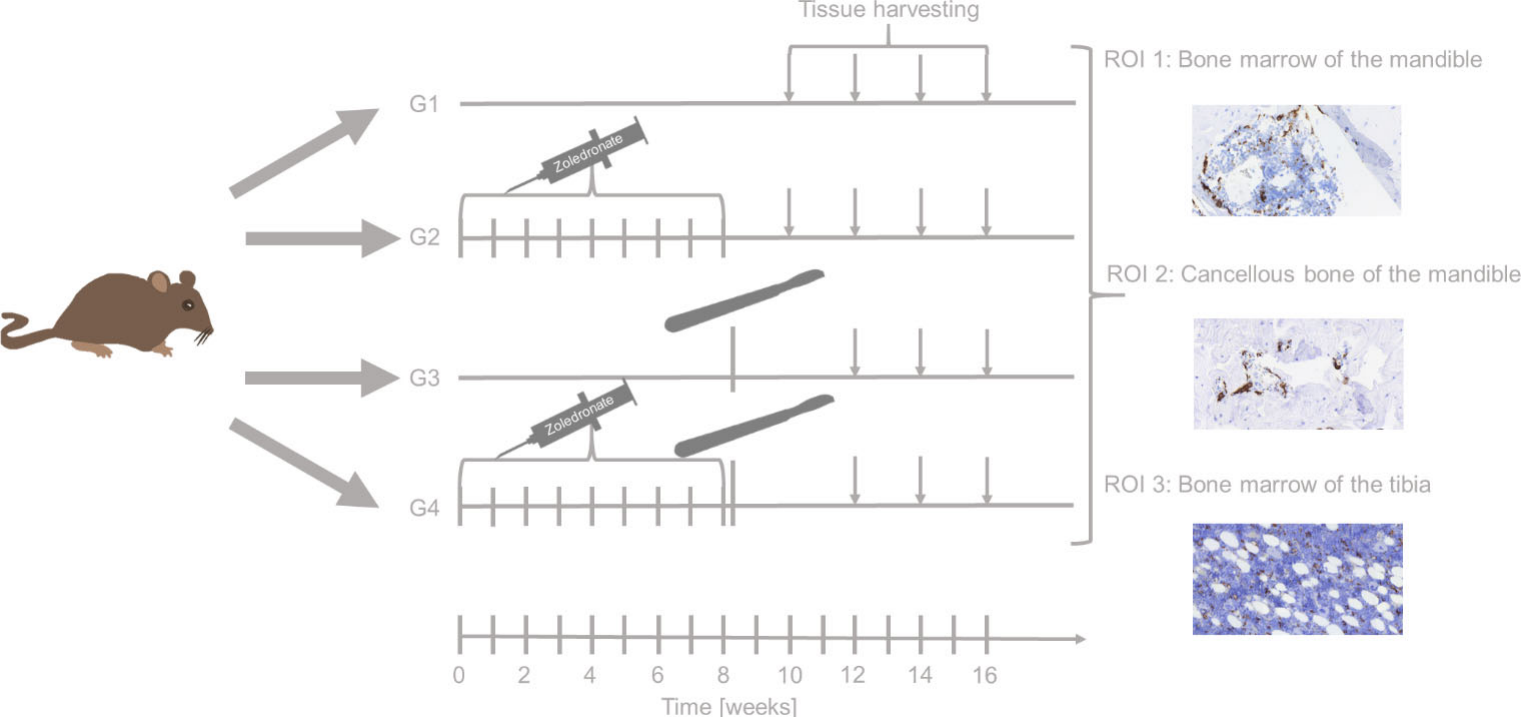
Study schedule. Rats from group (G) 2 and 4 were injected with zoledronate weekly for 8 weeks. After 8 weeks, surgical interventions (i.e, tooth extraction, tibia fracture followed by osteosynthesis) surgery was undertaken in rats from G3 and G4. Rats were euthanized after 8 (G1 and G2 only), 10, 12, and 16 weeks. Afterwards, tissue harvesting took place immediately. Tissue samples from the extraction socket (bone marrow and the cancellous bone of the mandible) as well as the repaired tibia fracture (bone morrow ot the tibia) were analyzed and further investigated by immunohistochemistry.

G2 (n = 32) and G4 (n = 20) received Zol (Zometa, Novartis, Basel, Switzerland) at a dose of 40 µg/kg body weight administered intraperitoneally (i.p.) as a weekly injection for 8 weeks. After this period, the right lower molar of the animals from G3 (n = 26) and G4 was extracted with forceps and the right tibia osteotomized followed by an immediate rigid osteosynthesis (8-hole 2.0 mini plate and 4 screws, Stryker, Kalamazoo, MI, US; see **Figure 1**). Surgical interventions were conducted under general anesthesia using 100 mg/kg of ketamine hydrochloride (Parke-Davis, Berlin, Germany) and 2.5 mg/kg of Xylocain (Bayer, Leverkusen, Germany) i.p. Postoperative analgesia was done using 2 mg/kg Buprenorphin (Temgesic, Essex Pharma, Munich, Germany) i.p. for the first and second postoperative days and further if necessary. G1 (n = 30) served as an untreated control group. At fixed time points (8, 10, 12 and 16 weeks after the start of the experiment for G1 and G2; 10, 12 and 16 weeks for G3 and G4) rats from each group were euthanized and tissue samples were taken from the extraction socket and the repaired tibia fracture on the right side. Moreover, samples were taken from the opposing jaw bone, i.e., the untreated left side for comparison. **Figure 2** illustrates the time schedule of the experiment. Dropouts (n = 12) were caused by animals that had to be sacrificed because of complications during surgery, i.e., anesthesiological complications, or postoperative infections.

### Tissue preparation

After the samples were obtained, they were fixed in formalin (4%) and embedded in paraffin. Following, the paraffin blocks were cut using a rotary microtome (Leica, RM2265, Wetzlar, Germany) with a slice thickness of 2 to 3 μm. After that, the sections were unfolded in a paraffin stretching bath (Medax, San Possidonio, Italy) at 43°C and mounted on Flex IHC Microscope Slides (DAKO, Glostrup, Denmark). Next, the slides were stored in a heating cabinet (IN30 incubator, Memmert, Schwabach, Germany) for 24 hours at a maximum of 58°C to achieve additional adhesion. Surgical specimens were analyzed by hematoxylin and eosin staining in order to microscopically assess the structures presented. Subsequently the prepared sections were processed for immunohistochemistry (IHC).

### Immunohistochemical staining

Immunohistochemical staining was performed on whole slides. Sections of rat spleen were included as a positive control with each run. In addition, tissue samples form the mandible or the tibia without application of the primary antibodies were used as negative controls.

Before staining, the paraffin-embedded slides were deparaffinized in xylene (Carl Roth, Karlsruhe, Germany; 3x for 15 min) and soaked in reducing concentrations of propanol (Carl Roth; 2x100%, 2x96%, 2x90% and 2x70%, each for 3 min) to rehydrate. The slides were soaked in distilled water for 2 min und then immersed in washing buffer (pH 7.6, DAKO S3006). In the next step, slides were boiled in citric acid buffer (pH 6.0, Thermo Fisher Scientific, Waltham, Massachusetts, US) at 100°C for 30 min to retrieve antigens und cooled down for 30 min at room temperature. Then, slides were immersed in washing buffer.

From this point, an automated slide stainer (Cytomation Autostainer plus, DAKO) was used. The slides were incubated with Peroxidase Blocking Solution 3% (DAKO, S2023) for 15 min to suppress endogenous peroxidase activity. Following, the slides were rinsed with washing buffer and were blocked in Proteinblock (DAKO, X0909) for 30 min. Liquid was blowed off the slides and they were incubated in the 100-fold diluted anti-CD68 antibody/100-fold diluted anti-CD163 antibody (anti-CD68: MCA341R; anti-CD163: MCA342R, both Bio-Rad Laboratories, Hercules, California, United States) in antibody diluent (DAKO, S2022). After a washing step, the slides were incubated with the secondary antibody (Solution A, DAKO, Kit LSAB 2, K0609) for 15 min. A washing step followed, after which the slides were incubated with Streptavidin-HRP-complex (Solution B; DAKO, Kit LSAB 2, K0609) at room temperature for 15 min. Slides were rinsed in washing buffer and developed in DAB solution for 10 min (Solution C; DAKO, Kit LSAB 2, K0609). Finally, slides were washed with washing buffer and distilled aqua.

Counterstaining was done rinsing the slides in haemalaun (DAKO, S3301) for 5 min, followed by rinsing them with running tab water. As a final step, the slides were mounted with Aquatex (Merck, Darmstadt, Germany).

### Quantitative immunohistochemical analysis

Slides were scanned with a Pannoramic 250 scanner (3DHISTECH, Budapest, Hungary) for further investigation. The immunohistochemical stainings were analyzed using the program ImageJ (National Institutes of Health (NIH), Maryland, US). Three visual fields per slide were investigated as regions of interest (ROIs). The ROIs were the following: ROI 1: bone marrow of the mandible, ROI 2: cancellous bone of the mandible, and ROI 3: bone marrow of the tibia.

First, the cells were assessed by staining, size, and form. Next, the cell count was determined by counting the number of positively stained cells per mm^2^ of the ROI. Following, the labeling index (LI) was calculated by dividing the number of positive cells by the overall number of cells counted in the ROI.

### Statistical analysis

Statistical analysis was performed using the Statistical Package for the Social Sciences 27.0 (SPSS, Chicago, IL, US). Median, standard deviation, and Mann-Whitney-U-test were used to determine differences between the groups. The box plot diagrams represent the median, interquartile range, minimum, and maximum. Two-sided adjusted p values ≤0.05 were considered statistically significant.

## Results

An animal experiment with 120 rats was performed in order to investigate the effect of Zol and surgical interventions on macrophage polarization in the craniofacial and extracranial skeleton. Tissue samples of 108 animals were available for the analyses. Dropouts (n = 12) were caused by animals that had to be sacrificed because of complications during surgery, i.e., anesthesiological complications, or postoperative infections.

**Figure 3:**
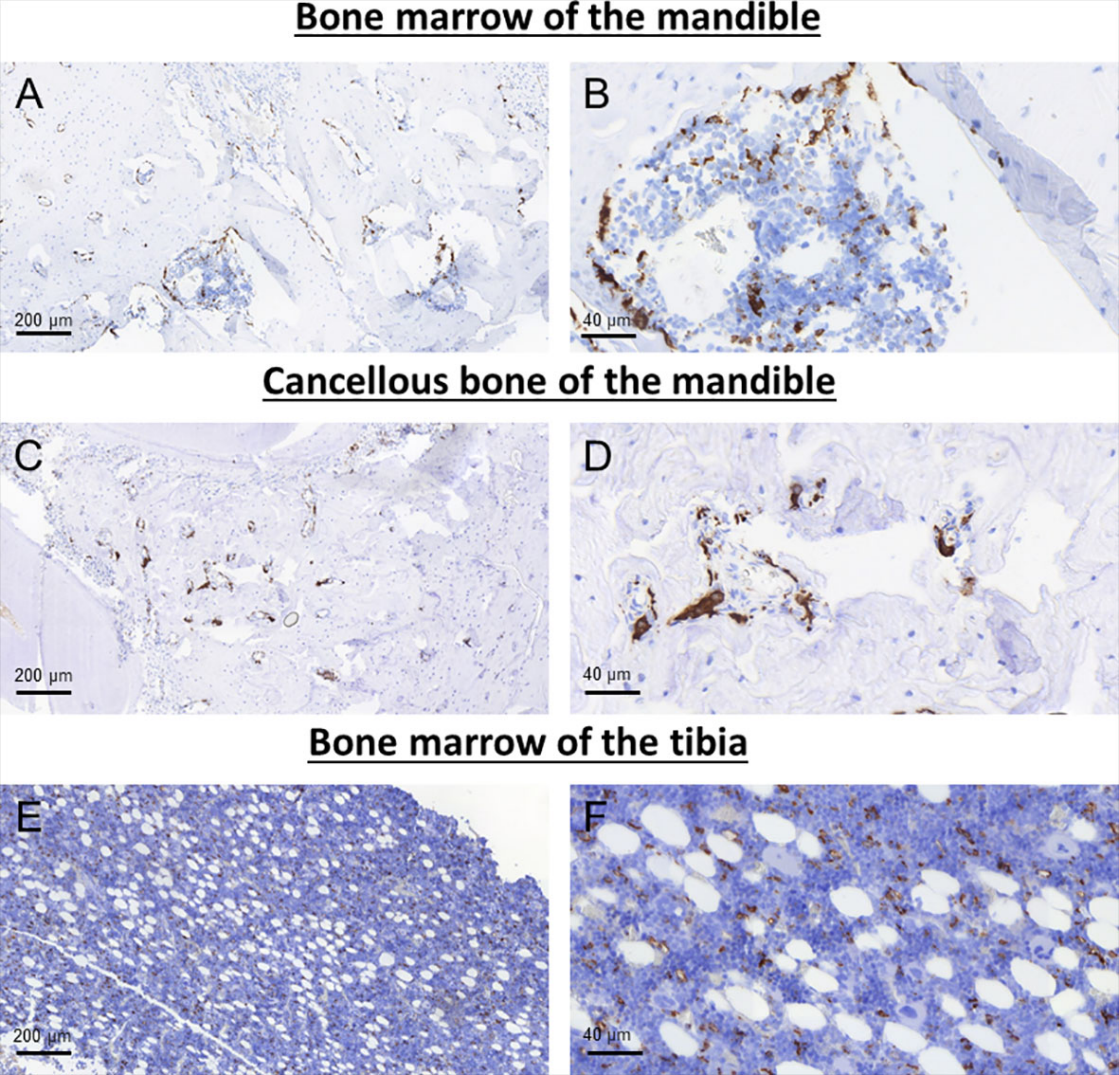
Representative images of CD68 immunohistochemical staining in the bone marrow **(A, B)** and cancellous bone of the mandible **(C, D)** as well as the bone marrow of the tibia **(E, F)**.

**Figure 4:**
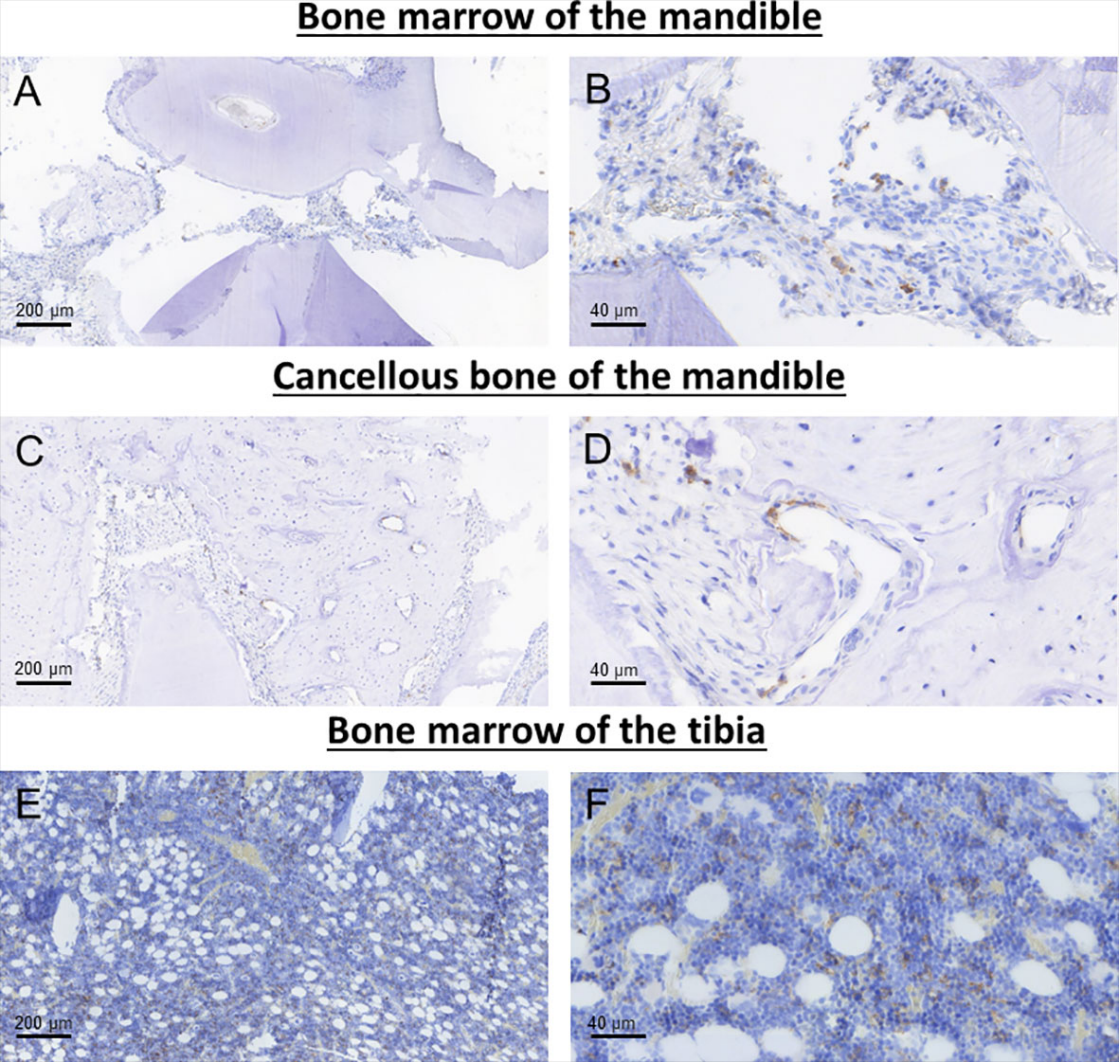
Representative images of CD163 immunohistochemical staining in the bone marrow **(A, B)** and cancellous bone of the mandible **(C, D)** as well as the bone marrow of the tibia **(E, F)**.

The staining pattern of the macrophage markers analyzed in this study (pan-macrophage marker CD68 and M2 macrophage marker CD163) was mainly membranous and cytoplasmatic. Representative micrographs of the staining patterns are shown in **Figures 3**, **4**.

Because there was no relevant correlation between the time of sacrifice and the measured macrophage infiltration and polarization, the time points 8, 10, 12, and 16 weeks after the start of the experiment were summarized for all analyses.

### Influence of zoledronate on the macrophage polarization in the bone marrow and the cancellous bone of the mandible

**Figure 5:**
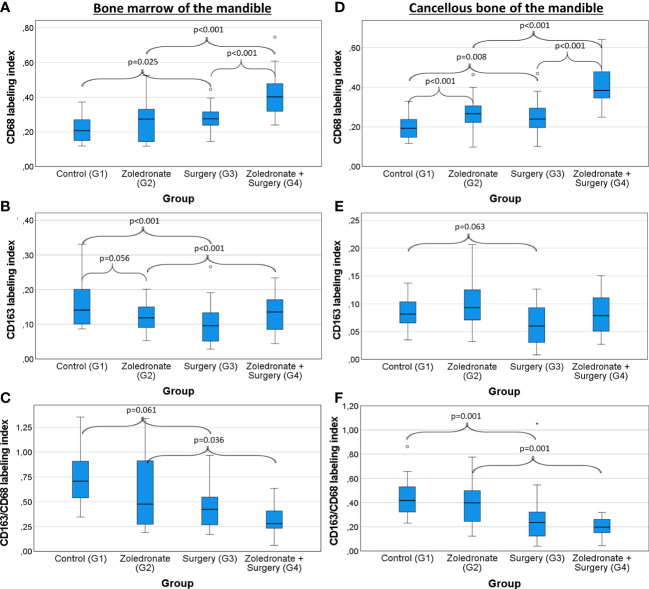
Influence of zoledronate treatment and surgical interventions on the macrophage polarization in the bone marrow and the cancellous bone of the mandible. Labeling index (LI) of **(A)** CD68^+^ cells, **(B)** CD163^+^ cells, and the **(C)** expression ratio of CD68 and CD163 (LI CD163/CD68) in the bone marrow of the jaw depending on the interventions. LI of **(D)** CD68^+^ cells, **(E)** CD163^+^ cells, and the **(F)** expression ratio of CD163 and CD68 (LI CD163/CD68) in the the cancellous bone of the tibia depending on the interventions. Rats from group (G) 1 were compared to those from G2 and G3. Rats from G4 were compared to those from G2 and G3. The box plot diagrams represent the median, interquartile range, minimum and maximum. For statistical analysis, the Mann-Whitney-U-test was used. Two-sided adjusted p values ≤0.05 were considered statistically significant. Statistically significant differences are marked with an asterisk.

Zol treatment (G2) enhanced the number of macrophages (LI CD68) in the mandible. However, a statistical significant difference was only reached in the cancellous bone (median 0.19 and 0.27, p<0.001; bone marrow: median 0.21 and 0.27, p=0.376; **Figures 5A, D**; **Table 1**). Moreover, Zol treatment reduced the LI of anti-inflammatory CD163^+^ macrophages in the bone marrow of the mandible (median 0.15 and 0.12, p=0.056; **Figure 5B**; **Table 1**). The ratio between CD163 and CD68 expression can be considered as an indicator of M2 polarization of macrophages. Overall, there was a decrease in CD163/CD68 ratio in the bone marrow of the mandible after Zol application, whereas it remained stable in the cancellous bone (bone marrow: median 0.71 and 0.48, p=0.102; cancellous bone: median 0.42 and 0.40, p=0.404; **Figures 5C, E**; **Table 1**). Results are shown in **Figure 5**; **Table 1**.

**Table 1:** Macrophage polarization in the bone marrow of the mandible, the cancellous bone of the mandible, and the bone marrow of the tibia.

LI	CD68			CD163			CD163/CD68		
	n	Median	SD	n	Median	SD	n	Median	SD
Bone marrow of the mandible									
G1	19	0.21	0.08	22	0.15	0.08	18	0.71	0.28
G2	23	0.27	0.12	20	0.12	0.04	20	0.48	0.36
G3	17	0.28	0.08	25	0.10	0.06	16	0.42	0.22
G4	16	0.40	0.13	16	0.14	0.05	14	0.28	0.16
p values of the comparison between the different treatment groups of rats									
G1 vs. G2 G1 vs. G3 G1 vs. G4 G2 vs. G4 G3 vs. G4	p=0.376 p=0.025* p<0.001* p<0.001* p<0.001*		p=0.056 p=0.001* p=0.421 p<0.001* p=0.116				p=0.102 p=0.001* p<0.001* p=0.036* p=0.179		
Cancellous bone of the mandible									
G1	26	0.19	0.05	20	0.09	0.03	20	0.42	0.16
G2	28	0.27	0.07	28	0.10	0.04	27	0.40	0.18
G3	20	0.24	0.09	18	0.06	0.04	18	0.24	0.23
G4	18	0.39	0.10	15	0.08	0.04	15	0.20	0.08
p values of the comparison between the different treatment groups of rats									
G1 vs. G2 G1 vs. G3 G1 vs. G4 G2 vs. G4 G3 vs. G4	p<0.001* p=0.008* p<0.001* p<0.001* p<0.001*		p=0.207 p=0.063 p=0.735 p=0.245 p=0.108				p=0.404 p=0.001* p<0.001* p=0.001* p=0.656		
Bone marrow of the tibia									
G1	22	0.16	0.05	22	0.13	0.05	22	0.90	0.23
G2	29	0.16	0.05	29	0.14	0.05	29	0.76	0.33
G3	13	0.19	0.07	13	0.10	0.10	13	0.63	0.33
G4	10	0.23	0.11	11	0.16	0.06	10	0.56	0.32
p values of the comparison between the different treatment groups of rats									
G1 vs. G2 G1 vs. G3 G1 vs. G4 G2 vs. G4 G3 vs. G4	p=0.321 p=0.091 p=0.070 p=0.022* p=0.186		p=0.924 p=0.243 p=0.930 p=0.473 p=0.303				p=0.068 p=0.031* p=0.025* p=0.059 p=0.784		

Shows the CD68 (pan-macrophage marker), CD163 (M2 macrophage marker), and the CD163/CD68 labeling index (LI) in tissue samples from the bone marrow of the mandible, the cancellous bone of the mandible and the bone marrow of the tibia. Macrophage populations were compared between the rats treated with zoledronate (group (G) 2), surgical interventions (i.e., tooth extraction and tibia fracture, G3), rats treated with both interventions (zoledronate + surgery, G4) and the rats which served as untreated control group (G1).

Values represent the median, standard deviation (SD), and p value. Statistical analyses were performed using the Mann-Whitney-U-test.

Asteriks represent statistical differences between groups (* indicates p value <0.05).

LI, labeling index; n, number of cases.

### Influence of tooth extraction on the macrophage polarization in the bone marrow and cancellous bone of the mandible

The CD68 LI increased significantly after tooth extraction in the jaw (G3; bone marrow: median 0.21 and 0.28, p<0.001; cancellous bone: 0.19 and 0.24, p=0.008; **Figures 5A, D**; **Table 1**). In addition, the CD163 LI decreased significantly in the bone marrow of the mandible (median 0.15 and 0.010, p=0.001; **Figure 5B**; **Table 1**), whereas statistical significance was narrowly missed in the cancellous bone (median 0.09 and 0.06, p=0.063; **Figure 5E**; **Table 1**). Accordingly, CD163/CD68 ratio was significantly decreased in the extraction socket (bone marrow: median 0.71 and 0.42, p=0.001; cancellous bone: median 0.42 and 0.24, p=0.001; **Figures 5C, F**; **Table 1**).

**Figure 6:**
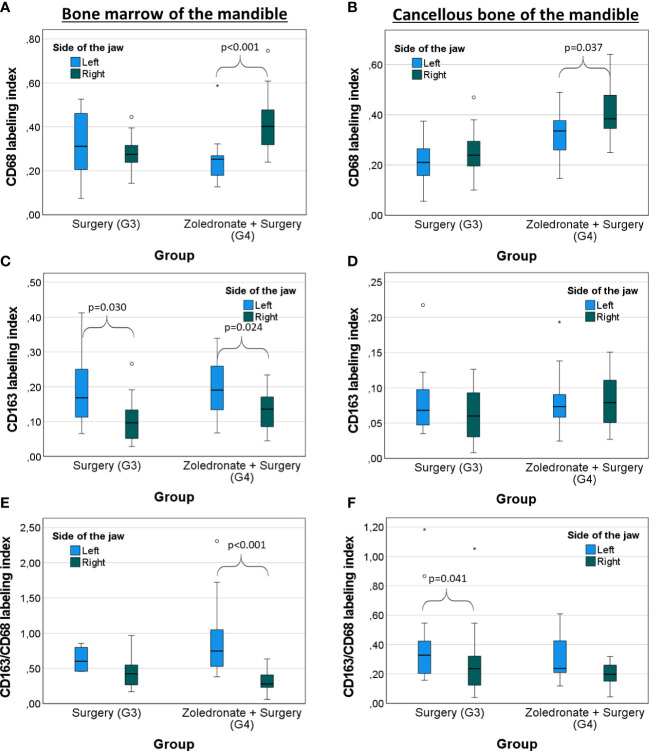
Comparison between both sides of the jaw bone of rats treated with surgery and combinatorial treatment with surgery and zoledronate treatment regarding the macrophage polarization. Surgical interventions were conducted on the right side whereas the left jaw side served as control. Labeling index (LI) of **(A)** CD68^+^ cells, **(B)** CD163^+^ cells, and the **(C)** expression ratio of CD68 and CD163 (LI CD163/CD68) in the bone marrow of the mandible depending on the interventions. LI of **(D)** CD68^+^ cells, **(E)** CD163^+^ cells, and the **(F)** expression ratio of CD68 and CD163 (LI CD163/CD68) in the cancellous bone of the mandible depending on the interventions. The box plot diagrams represent the median, interquartile range, minimum, and maximum. For statistical analysis, the Mann-Whitney-U-test was used. Two-sided adjusted p values ≤0.05 were considered statistically significant. Statistically significant differences are marked with an asterisk.

Comparing the operated and the non-operated side of the jaw bone in rats which underwent surgery, we observed a decrease of anti-inflammatory CD163^+^ macrophages in the bone marrow of the operated side compared to the non-operated side (LI; median: 0.17 and 0.10 p=0.030; **Figure 6**; **Tables 2**; **3**).

**Table 2:** Comparison of the macrophage polarization in the bone marrow of the mandible between the operated and the unoperated side.

LI	CD68			CD163			CD163/CD68		
	n	Median	SD	n	Median	SD	n	Median	SD
Rats treated with surgical interventions (G3)									
Operated side (right)	17	0.28	0.08	20	0.10	0.06	16	0.42	0.22
Unoperated side (left)	11	0.31	0.16	9	0.17	0.13	4	0.60	0.20
p values									
Operated side vs. non operated side	p=0.430			p=0.030*			p=0.131		
Rats treated with zoledronate and surgical interventions (G4)									
Operated side (right)	16	0.40	0.13	16	0.14	0.05	14	0.28	0.16
Unoperated side (left)	15	0.25	0.11	15	0.19	0.08	14	0.75	0.54
p values									
Operated side vs. non operated side	p<0.001*			p=0.024*			p=0.001*		

Shows the CD68 (pan-macrophage marker), CD163 (M2 macrophage marker), and the CD163/CD68 labeling index (LI) in the bone marrow of the mandible from rats treated with surgical interventions (i.e., tooth extraction and tibia fracture, G3) and rats treated with zoledronate application and surgical intervention (G4). The macrophage populations were compared between the operated side of the jaw, i.e., the right side, and the unoperated side, i.e., the left side.

Values represent the median, standard deviation (SD), and p value. Statistical analyses were performed using the Mann-Whitney-U-test.

Asteriks represent statistical differences between groups (* indicates p value <0.05).

LI, labeling index; n, number of cases.

**Table 3:** Comparison of the macrophage polarization in cancellous bone of the mandible between the operated and the unoperated side.

LI	CD68			CD163			CD163/CD68		
	n	Median	SD	n	Median	SD	n	Median	SD
Rats treated with surgical interventions (G3)									
Operated side (right)	16	0.21	0.08	16	0.07	0.05	13	0.33	0.30
Unoperated side (left)	13	0.34	0.09	14	0.08	0.04	13	0.24	0.16
p values									
Operated side vs. non operated side	p=0.161			p=0.255			p=0.041*		
Rats treated with zoledronate and surgical interventions (G4)									
Operated side (right)	20	0.24	0.09	18	0.06	0.04	18	0.24	0.23
Unoperated side (left)	18	0.39	0.10	15	0.08	0.04	15	0.20	0.08
p values									
Operated side vs. non operated side	p=0.037*			p=0.983			p=0.240		

Shows the CD68 (pan-macrophage marker), CD163 (M2 macrophage marker), and the CD163/CD68 labeling index (LI) in the cancellous bone of the mandible from rats treated with surgical interventions (i.e., tooth extraction and tibia fracture, G3) and rats treated with zoledronate application and surgical intervention (G4). The macrophage populations were compared between the operated side of the jaw, i.e., the right side, and the unoperated side, i.e., the left side.

Values represent the median, standard deviation (SD), and p value. Statistical analyses were performed using the Mann-Whitney-U-test.

Asteriks represent statistical differences between groups (* indicates p value <0.05).

LI, labeling index; n, number of cases.

### Influence of tooth extraction on the macrophage polarization in zoledronate-treated rats

In rats which got Zol treatment before tooth extraction (G4), we observed a significant increase of the overall macrophages in the mandible compared to the untreated rats (LI CD68; bone marrow: median 0.28 and 0.40, p<0.001 and cancellous bone: median 0.24 and 0.39, p<0.001; **Figures 5A, D**; **Table 1**). On the contrary, the LI of anti-inflammatory macrophages (CD163^+^) remained stable in the jaw (bone marrow: 0.10 and 0.14, p=0.116; cancellous bone: median 0.06 and 0.08, p=0.108; **Figures 5B, E**; **Table 1**), indicating an environment of heightened pro-inflammatory response in the mandible compared to the rats which underwent surgery only without BP application. Comparing the macrophage populations in the jaw bone of rats from G4 to those of rats from G2 and G3, the CD68 LI was significantly higher in G4 (BP and tooth extraction) than in G2 (BP without tooth extraction) (bone marrow: 0.27 and 0.40, p<0.001; cancellous bone: 0.27 and 0.39, p<0.001; **Figures 5A, D**; **Table 1**) and G3 (bone marrow: 0.28 and 0.40, p<0.001; cancellous bone: 0.24 and 0.39, p<0.001; **Figures 5A, D**; **Table 1**). In addition, there were significantly less anti-inflammatory macrophages in the mandible of rats from G4 than in G2 (CD163 LI, bone marrow: 0.12 and 0.14, p<0.001; cancellous bone: 0.10 and 0.08, p=0.245; CD163/68 LI, bone marrow: 0.48 and 0.28, p=0.036; cancellous bone: 0.40 and 0.20, p=0.001; **Figures 5B, C, E, F**; **Table 1**). This decrease of anti-inflammatory macrophages was only seen on the operated side of the jaw, whereas the macrophage phenotypes on the non-operated side did not change (p>0.05; **Figure 6**; **Tables 2**; **3**).

### Comparison of the macrophage polarization between the bone marrow and the cancellous bone of the mandible

Comparing the bone marrow and the cancellous bone of the mandible of rats from G1 (control group), we observed a significantly higher CD163 LI (median 0.15 and 0.09, p<0.001; **Table 4**) in line with a significantly higher CD163/CD68 ratio in the bone marrow (LI; median 0.71 and 0.42, p<0.001; **Table 4**). After Zol application (G2; median 0.12 and 0.09, p<0.001; **Table 4** **)**, surgery (G3; median 0.10 and 0.06, p=0.046; **Table 4**) or combination of both the CD163 LI (median 0.14 and 0.08, p=0.010; **Table 4**) was still significantly higher in the bone marrow. In addition, the CD163/CD68 ratio was significantly higher in the bone marrow of the rats undergoing surgery (median 0.42 and 0.24, p=0.012; **Table 4**) and combinatorial treatment of Zol application and surgery (median 0.28 and 0.20, p=0.029; **Table 4**) than in the cancellous bone. However, there was no significant difference regarding the CD163/CD68 ratio after Zol application (median 0.48 and 0.42, p=0.127; **Table 4**). Results of the comparative analysis are shown in **Table 4**.

**Table 4:** Macrophage polarization in rats treated with zoledronate (G2), surgical interventions (G3), both interventions (G4), and without any treatment (G1).

LI	CD68			CD163			CD163/CD68		
	n	Median	SD	n	Median	SD	n	Median	SD
Untreated controls (G1)									
M-BM	19	0.21	0.08	22	0.15	0.08	18	0.71	0.28
M-CB	26	0.19	0.05	20	0.09	0.03	20	0.42	0.16
T-BM	22	0.16	0.05	22	0.13	0.05	22	0.90	0.23
p values of the comparison between the regions of interest									
M-BM vs. M-CB M-BM vs. T-BM M-CB vs. T-BM	p=0.316 p=0.026* p=0.038*			p<0.001* p=0.979 p=0.006*			p<0.001* p=0.029* p<0.001*		
Rats treated with zoledronate application (G2)									
M-BM	23	0.27	0.12	20	0.12	0.04	20	0.48	0.36
M-CB	26	0.19	0.05	20	0.09	0.03	20	0.42	0.16
T-BM	29	0.16	0.05	29	0.14	0.05	29	0.76	0.33
p values of the comparison between the regions of interest									
M-BM vs. M-CB M-BM vs. T-BM M-CB vs. T-BM	p=0.496 p=0.012* p<0.001*			p=0.046* p=0.802 p=0.303			p=0.127 p=0.077 p=0.002*		
Rats treated with surgical interventions (G3)									
M-BM	17	0.28	0.08	25	0.10	0.06	16	0.42	0.22
M-CB	20	0.24	0.09	18	0.06	0.04	18	0.24	0.23
T-BM	13	0.19	0.07	13	0.10	0.10	13	0.63	0.33
p values of the comparison between the regions of interest									
M-BM vs. M-CB M-BM vs. T-BM M-CB vs. T-BM	p=0.223 p=0.002* p=0.017*			p=0.020* p=0.357 p=0.003*			p=0.012* p=0.016* p<0.001*		
Rats treated with zoledronate application and surgical interventions (G4)									
M-BM	16	0.40	0.13	16	0.14	0.05	14	0.28	0.16
M-CB	18	0.39	0.10	15	0.08	0.04	15	0.20	0.08
T-BM	10	0.23	0.11	11	0.16	0.06	10	0.56	0.32
p values of the comparison between the regions of interest									
M-BM vs. M-CB M-BM vs. T-BM M-CB vs. T-BM	p=0.945 p=0.003* p=0.002*			p=0.010* p=0.490 p=0.012*			p=0.029* p=0.002* p<0.001*		

Shows the CD68 (pan-macrophage marker), CD163 (M2 macrophage marker), and the CD163/CD68 labeling index (LI) in tissue samples from the bone marrow of the mandible (M-BM), the cancellous bone of the mandible (M-CB), and the bone marrow of the tibia (T-BM).

Values represent the median, standard deviation (SD), and p value. Statistical analyses were performed using the Mann-Whitney-U-test.

Asteriks represent statistical differences between groups (* indicates p value <0.05).

LI, labeling index; n, number of cases.

### Influence of zoledronate and surgical interventions on the bone marrow of the tibia

**Figure 7:**
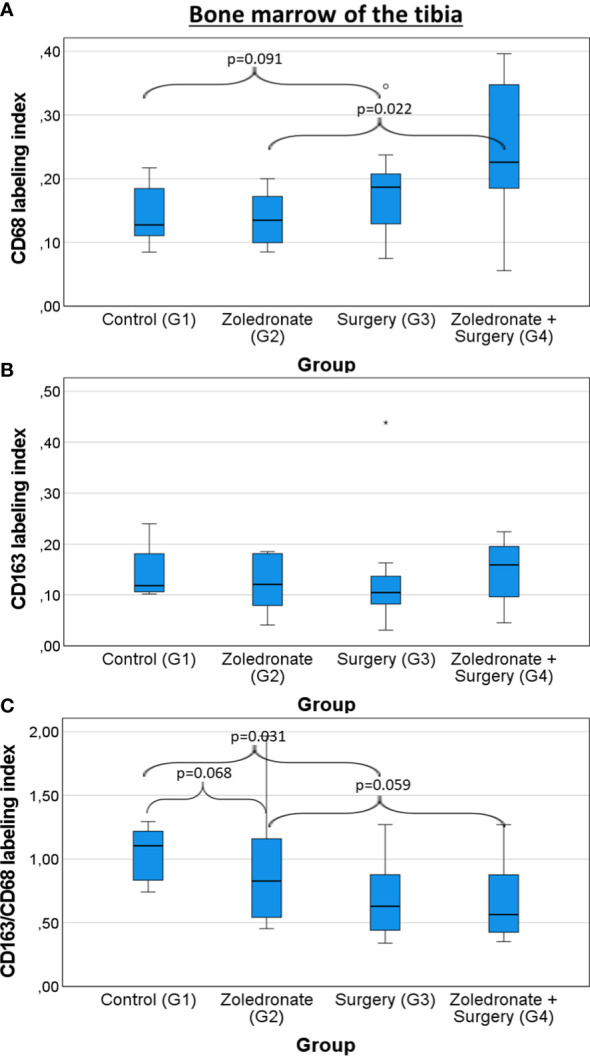
Influence of zoledronate treatment and surgical interventions on the macrophage polarization in the bone marrow of the tibia. Labeling index (LI) of **(A)** CD68^+^ cells, **(B)** CD163^+^ cells, and the **(C)** expression ratio of CD68 and CD163 (LI CD163/CD68) in the bone marrow of the tibia depending on the interventions. Rats from group (G) 1 were compared to those from G2 and G3. Rats from G4 were compared to those from G2 and G3. The box plot diagrams represent the median, interquartile range, minimum, and maximum. For statistical analysis, the Mann-Whitney-U-test was used. Two-sided adjusted p values ≤0.05 were considered statistically significant. Statistically significant differences are marked with an asterisk.

In contrast to the jaw, the CD68 LI (median 0.16 and 0.16, p=0.321; **Figure 7A**; **Table 1**) and the CD163 LI (median 0.13 and 0.14, p=0.924; **Figure 7**; **Table 1**) remained stable in the bone marrow of the tibia after Zol application. When surgery was conducted, the CD68 LI significantly increased in Zol-treated rats compared to the rats without Zol treatment (median 0.16 and 0.23, p=0.022; **Figure 7A**; **Table 1**). Likewise, there was a decrease in the CD163/CD68 ratio, but the difference between both groups did not reach significance (median 0.76 and 0.56, p=0.059; **Figure 7C**; **Table 1**). Like in the mandible, the CD163/CD68 ratio decreased significantly in the tibia after surgery compared to the control group (median 0.90 and 0.63, p=0.031; **Figure 7C**; **Table 1**), indicating a postoperative environment dominated by pro-inflammatory immune cells.

### Comparison of the macrophage polarization between the jaw bone and the tibia

The macrophage composition differed significantly among the tibia and the mandible. Without any intervention (G1), the CD68 LI was significantly higher in the bone marrow (median 0.21 and 0.16, p=0.026; **Table 4**) and the cancellous bone of the mandible (median 0.19 and 0.16, p=0.038; **Table 4**) than in the tibia. In addition, the CD163/CD68 ratio was significantly lower in the bone marrow (median 0.71 and 0.90, p=0.029; **Table 4**) and the cancellous bone of the mandible (median 0.42 and 0.90, p<0.001; **Table 4**), indicating a pro-inflammatory environment in the jaw bone compared to the tibia.

After all interventions the pattern remained (G2-G4): The CD68 LI was significantly higher in the bone marrow (p<0.05; **Table 4**) and cancellous bone of the mandible (p<0.05; **Table 4**) than in the tibia. After surgery and the combined treatment with Zol and surgery, the CD163/CD68 ratio was still significantly lower in the cancellous bone and the bone marrow of the mandible than in the tibia (p<0.05; **Table 4**). Results of the comparative analysis are shown in **Table 4**.

## Discussion

Recent evidence indicates that macrophages might play a pivotal role in MRONJ pathogenesis (21). The understanding of their polarization and therefore composition depending on Zol application and surgical interventions (i.e., tooth extraction) might help to improve current prevention and therapy of MRONJ. Differences between the craniofacial and extracranial skeleton regarding the immune cell composition might be an additional explanation for the unique predisposition of the jaw bone for MRONJ. Therefore, we compared the macrophage polarization in the mandible to those of a long bone belonging to the extracranial skeleton, i.e., the tibia. In addition, understanding the influence of BPs on bone macrophages might improve our understanding of the anti-metastatic effects of BPs.

In the present study, we demonstrated that Zol treatment as well as tooth extraction upregulates the overall number of macrophages in the jaw bone. Meanwhile, the percentage of anti-inflammatory macrophages (CD163^+^ M2 macrophages) decreased. The most significant decrease of anti-inflammatory macrophages was observed after combinatorial treatment of tooth extraction and previous Zol application.

The influence of Zol application on macrophage polarization was investigated previously. However, to this point, studies about the effect of tooth extraction on macrophage polarization in the mandible are missing.

In the same Wistar rat model, our group previously demonstrated a significant shift of macrophage polarization toward M1 in skin, spleen, and lung tissue of rats treated with BPs and surgical interventions (i.e., tooth extraction and tibia fracture) (45). In addition, Kaneko et al. investigated the polarization of human monocytic THP-1 cells to macrophage like cells *in vitro* depending on Zol application. In line with our results, they observed that Zol treatment enhanced LPS-induced M1 macrophage polarization. On the contrary, M2 polarization was not affected. Kaneko et al. attributed the macrophage polarization toward M1 after Zol treatment to the upregulation of the NLRP3 inflammasome and therefore the secretion of Interleukine-1β (46). In accordance with our findings, Hajano et al. found a decreased number of M2 macrophages after Zol application in a mouse model (47).

Zhu et al. investigated the effect of BPs on macrophage polarization and its association with the toll-like receptor (TLR)-4 signaling pathway *in vitro* (bone marrow-derived macrophages cell culture) and *in vivo* (mouse model) (48). The TLR-4 pathway is a pro-inflammatory pathway which plays an important role in inducing the innate immune response and thereby, the release of pro-inflammatory cytokines (49). Zhu et al. observed an elevated TLR-4 expression in macrophages after Zol application, resulting in an enhanced M1 macrophage polarization and decreased M2 macrophage polarization. Interestingly, an inhibition of the TLR-4 pathway suppressed the secretion of pro-inflammatory cytokines by macrophages and thereby, the prevalence of M1 macrophages (48). Inhibitors of the TLR-4 pathway have been investigated in varius diseases and showed positive effects aginst sepsis (50), cerebral ischemia (51), and inflammatory bowel disease (52).

Moreover, several researchers investigated the macrophage polarization in human MRONJ lesions: Our group previously observed a significantly increased macrophage infiltration and a shift toward M1 macrophages in jaw bone affected by MRONJ (53).

Hoefert et al. observed a lower M2/M1 macrophage ratio in the MRONJ lesions compared with other infections of the jaw bone and concluded that imbalanced polarization of macrophages induced by BPs may be linked to the development of MRONJ. However, in contrast to our results, Hoefert et al. did not observe a change regarding the macrophage phenotypes in patients receiving BPs but not showing a MRONJ at that point (21).

Altogether, we propose the hypothesis that the decrease in M2 macrophages might already exist after Zol treatment and tooth extraction and not only after development of MRONJ. Since patients receiving antiresorptive therapy often have multiple medical comorbidities, the advantage of our study might have been that we were able to conduct our study under standardized conditions without any concomitant diseases of the participants.

In addition, the more pro-inflammatory (i.e., anti-tumoral) microenvironment after Zol application supports the hypothesis of an anti-tumoral and anti-metastatic effect induced by BPs. BPs might promote anti-tumoral activity in the tumor microenvironment, leading to an inhibition of cancer progression (39, 39). Future clinical trials are needed to investigate the underlying mechanisms and the effect of an additional treatment with BPs in patients with different types of cancer.

The importance of tooth extraction in enhancing a pro-inflammatory environment in the tooth extraction socket was verified by comparing the operated- and non-operated side of the jaw bone in Zol-treated rats. It is well accepted that tooth extraction is a major risk factor for the development of MRONJ (54).

A transient inflammatory phase is crucial for bone formation and remodeling after tooth extraction. The physiologic inflammatory response is dominated by M1 macrophages at first, and secondly by M2 macrophages (55). Kang et al. investigated the macrophage phenotypes involved in delayed socket healing in patients with diabetes mellitus type 2. They observed an impaired shift from M1 to M2 macrophages with a sustained secretion of M1-associated pro-inflammatory cytokines, leading to prolonged inflammation. The same mechanism might contribute to the development of MRONJ after Zol application. Tooth extraction in Zol-treated rats might further increase the inflammatory cytokine levels and therefore might promote MRONJ. This emphasizes the prevention of periodontal and periapical infections in MRONJ patients in order to avoid tooth extractions. However, the risk of developing MRONJ in patients receiving BPs is significantly lower, when tooth extraction is performed according to established guidelines including primary wound closure (56).

Altogether, we suggest that Zol, directly or indirectly, creates an environment of heightened pro-inflammatory response and reduced regenerative potential indicated by the reduced M2 polarization of macrophages. Surgical interventions like tooth extraction might further promote this effect. The combination of both might cause or contribute to tissue damage and attenuated wound healing and tissue regeneration. A shift from M1 to M2 macrophages and therefore a restoring of a balanced M1/M2 ratio might reduce the risk of developing a MRONJ and might improve treatment options. However, the underlying mechanisms of the macrophage shifts are not certainly understood, calling for further investigation.

The infiltrating macrophage phenotypes were compared between the cancellous bone and the bone marrow of the mandible in order to investigate the inflammatory response in both bone compartments after Zol application and tooth extraction. However, to date, the composition of macrophages has not been previously compared between both compartments. Our results indicate a more anti-inflammatory environment in the bone marrow of the mandible and a more pro-inflammatory environment in the cancellous bone in the mandible. This relation remained after Zol application and tooth extraction. Therefore, we suggest that future research should focus on strategies targeting the pro-inflammatory environment in the cancellous bone in the mandible.

In the next step, we compared the infiltrating macrophage phenotypes in the mandible and the tibia. Despite various reports on the role of macrophages in bone healing, a comparative study on the macrophage phenotypes between the mandible and the extracranial skeleton has not been conducted.

In general, there was a higher number of macrophages in the mandible compared to the tibia. Additionally, the number of anti-inflammatory M2 macrophages was significantly lower in the jaw, even without any interventions, thus pointing to an enhanced pro-inflammatory environment in the jaw compared to the extracranial skeleton. In contrast to the jaw, the LI of the overall and anti-inflammatory M2 macrophages in the tibia remained stable after Zol application. However, when surgery was conducted, the overall number of macrophages significantly increased in both bones of Zol-treated rats. Like in the mandible, the LI of M2 macrophages decreased significantly in the tibia after surgery, indicating a postoperative increase of pro-inflammatory macrophages and a reduced regenerative potential in both bone types after surgery.

The results of the macrophage composition in the tibia are in line with that of Nikovics et al., who found a macrophage population dominated by the M2 macrophage phenotype in a femur fracture in a rat model. However, the macrophage population was dominated by the M2 phenotype (57). Moreover, an increased number of M1 macrophage and consequently an upregulation of inflammatory cytokines was also previously described to promote bone cell apoptosis and thereby accelerated femoral head necrosis (58). All in all, our results indicate fundamental immunological differences between the jaw bone and the tibia (craniofacial vs. extracranial bone). As described previously, the decrease of M2 macrophages associated with a more pro-inflammatory microenvironment in the mandible might impair the mechanism of physiologic wound healing after tooth extraction and could potentially play a role in the initiation or perpetuation of MRONJ. Moreover, it might be a reason for the unique predisposition of the jaw bones for MRONJ.

## Limitations of this study

This study has some limitations. We chose a relative low dosing of Zol to avoid a direct toxic effect, which was previously described (59). Comparable studies chose a higher dosing (60, 61). However, to this point, there is no clear evidence indicating which kind dose of BPs in rat models best mimics human conditions.

In addition, the markers employed to identify different macrophage phenotypes differs (e.g., CD206 for M2 macrophages (62)). Given the heterogeneity of macrophages, further studies on their various subtypes are necessary. Moreover, the M1/M2 paradigm is an attempt to rationally categorize cells of high plasticity, but might be insufficient to meet the demands of detailed mechanistic explorations (63).

The phenotypes of macrophages and their functions might differ between rats and humans. However, the selected macrophage markers were successfully used in previous human studies and animal experiments (45, 53).

## Conclusion

The overall number of macrophages was significantly higher in the mandible than in the tibia. In addition, the percentage of anti-inflamamtory M2 macrophages was significantly higher in the mandible. Zol application did not influence the macrophage polarization in the tibia, whereas it did in the jaw. This might be an additional reason for the unique predisposition for MRONJ in the jaw bones. In addition, the changes in macrophage polarization after Zol application and tooth extraction in the jaw might contribute to reduced tissue regeneration and therefore, the pathogenesis of MRONJ. Further studies are needed to delineate the mechanisms and specify the contributions of the various macrophage phenotypes to MRONJ pathogenesis. Targeting macrophages might represent an attractive therapeutic target to prevent MRONJ and improve therapy in the future. Especially, targeting the TLR-4 pathway might be a promosing approach.

In addition, the more pro-inflammatory microenvironment after Zol application supports the hypothesis of an macrophage mediated anti-tumoral and anti-metastatic effect induced by BPs. Future clinical trials are needed to investigate the underlying mechanisms and the effect of an additional treatment with BPs in patients with different types of cancer.

## Data availability statement

The raw data supporting the conclusions of this article will be made available by the authors, without undue reservation.

## Ethics statement

The animal study was reviewed and approved by Regierung von Mittelfranken.

## Author contributions

A-KS contributed to the statistical assessment, interpreted the data and wrote the manuscript. FW formulated the hypothesis, applied for grant support (DFG), initiated the study and critically reviewed the manuscript. MW contributed to the hypothesis, conducted the study, performed surgery and tissue harvesting, interpreted the data and contributed relevantly to the manuscript. RP conducted the study, performed surgery and tissue harvesting, interpreted the data and critically reviewed the manuscript. JR supervised the immunohistochemical staining, was involved in the assessment of the stainings and critically reviewed the manuscript. MM, TM and LE performed the immunohistochemical staining and contributed to the statistical assessment. CG supervised the digital scanning of the slides and was involved in the assessment of the stainings. LT, RL and MK contributed to the discussion and critically reviewed the manuscript. All authors have read and agreed to the published version of the manuscript.
